# Education Research: Neuropalliative Care Education: A National Survey of Neurology Program and Fellowship Directors

**DOI:** 10.1212/NE9.0000000000200364

**Published:** 2026-09-03

**Authors:** Eileen Harrigan, Arielle M. Kurzweil, Maureen I. Ekwebelem, Daniel Shalev, Nuri Jacoby

**Affiliations:** 1 Department of Neurology, NYU Langone Health, New York, NY;; 2 Department of Medicine, New York Presbyterian-Weill Cornell Medical College, New York, NY; and; 3 Department of Neurology, Maimonides Medical Center and SUNY Downstate Health Sciences University, New York, NY.

## Abstract

**Background and Objectives:**

Patients with neurologic disease have a variety of unmet palliative care needs. Both the Accreditation Council for Graduate Medical Education and Academy of Neurology have emphasized the need for neuropalliative care education in recent years. Neurology training programs have widely variable practices in teaching neuropalliative care. This study aims to characterize current practices in providing neuropalliative care education across graduate neurology training and to identify barriers to its delivery.

**Methods:**

We surveyed program directors (PDs) of neurology residency and fellowship training programs in the United States regarding their current methods of teaching neuropalliative care topics, perceived importance of these topics, relevant subspecialty resources, and perceived barriers to providing neuropalliative care education.

**Results:**

Our survey was emailed to 777 PDs and fellowship directors. In total, 118 responses were included in the analytic sample (response rate 15.2%). Program directors generally perceived neuropalliative care education as important, with a median rank of 7 of 10. 91% of adult neurology residency programs and 66% of fellowship programs included palliative care topics in their didactics curricula. Topics emphasized varied depending on the training program's subspecialty. Respondents highlighted barriers to implementing neuropalliative care training, including inadequate trainee and faculty time and lack of adequate teaching faculty.

**Discussion:**

Neurology training programs continue to exhibit considerable variability in palliative care education curricula. Although most programs reported a presence of palliative care in their institutions, variability exists in the amount of palliative care education taught, the topics emphasized, and the teaching methods. Notably, there is an opportunity for fellowship programs to increase the amount of neuropalliative care topics taught in their curricula. Neurology residency and fellowship directors may benefit from using existing neuropalliative care curricula to enhance this area of education, partnering with local non-neurology faculty to teach palliative topics, and integrating palliative care topics into subspecialty training.

## Introduction

Palliative care is specialized medical care that aims to improve the quality of life for people living with serious illness through symptom relief, psychosocial and spiritual support, and assistance with complex medical decision making.^1^ Neurologic disorders are among the leading causes of death and disability worldwide and are often characterized by prolonged illness trajectories, unpredictable progression, and profound effects on patients' function, relationships, and identity.^2-4^ Patients with neurologic disease and their care partners are known to have needs in multiple domains of palliative care, including symptom management, advance care planning, hospice enrollment, and caregiver support.^5,6^ Many of these needs are often suboptimally addressed in traditional models of neurologic care delivery. Palliative care specialists help address these needs, although they may lack familiarity with certain neurologic symptoms, rare diagnoses, and subspecialized management.^7^ The Accreditation Council for Graduate Medical Education (ACGME) and the American Academy of Hospice and Palliative Medicine do not have specific recommendations or milestones for neurologic education in palliative care fellowships, so training and comfort level may be inconsistent when caring for patients with serious or complex neurologic disease.^8^ In addition, a shortage of palliative care specialists as well as concerns over burnout and program sustainability may limit the availability of this type of care in the future.^9^ As our population ages, the prevalence of severe neurologic illness, including stroke, neurodegenerative disease, Parkinson disease, and dementia, is expected to rise, which will further expand the need for clinicians capable of addressing the needs of these patients.^10-12^

Neuropalliative care is an emerging subspecialty aimed at addressing the needs of patients and families affected by neurologic disease. Neuropalliative care education has been repeatedly highlighted as a priority for advancing the quality of neurologic care and moving this field forward. The ACGME recommended palliative care training for all neurology residents in 2019.^13^ In 2022, the American Academy of Neurology (AAN) issued a position statement on neuropalliative care needs and highlighted deficits in neuropalliative care education, emphasizing the need to “identify the best educational approaches to empower neurologists to practice effective primary palliative care.”^14^ In the setting of a palliative care resource shortage, educating neurologists to deliver primary palliative care, defined as palliative care delivered by nonpalliative care–trained clinicians, is particularly urgent.

Despite the clinical need and widespread recognition of the importance of palliative care in neurology, training programs in the United States have curricula that are highly variable and, at times, absent of primary palliative care skills.^15,16^ Similarly, the range of palliative care needs varies considerably by neurologic subspecialty from breaking bad news or palliative extubation in the neuro-ICU to complex symptom management and advance care planning in movement disorders and neurodegenerative disease. Some topics, such as pain or symptom management, may be best taught in a traditional didactic format. By contrast, communication skills, palliative extubation, and other skills may be best taught by observation, simulation, or role-play scenarios.^16-20^ Efforts have been made to develop and disseminate neuropalliative care curricula such as EPEC-N, communication workshops, communication coaching, and neuropalliative fellowships, but uptake, current use, and barriers to use of these resources in neurology training programs are unknown.^19,21-23^ Neurology residency program leaders' perspectives on neuropalliative care education needs, barriers to implementation, available resources, and priorities for improvement have not been assessed since 2018, and neurology subspecialty fellowship director perspectives are unknown.^15^

To better understand current neuropalliative care education models and practices and to identify gaps and opportunities for improvement, we surveyed program directors (PDs) of neurology residency and fellowship programs. In this article, we describe the current state of neuropalliative care education as reported by these PDs and identify areas for improvement based on their responses.

## Methods

### Survey Design

Using Artino's 7-step process for educational survey design,^24^ we developed a survey to assess the presence, structure, and perceived importance of palliative care training in neurology residency and fellowship programs. Existing qualitative data on neuropalliative care training needs were available from previous studies,^25,26^ and our team had previously developed related instruments in psychiatry and geriatrics education.^25,26^ On this basis, we did not conduct de novo stakeholder interviews; instead, we drew on the existing literature and our group's experience in palliative care, neurology, and neuropalliative care education.^22^ Author DS (a psychiatrist and palliative care physician/scientist) developed an initial survey draft (Artino step 4), which then underwent expert validation through review, validation, and discussion/cognitive interviews (steps 5 and 6) with authors N. Jacoby, A.M. Kurzweil (both neurology physician/educators), and E. Harrigan (a neurologist, palliative care physician, and neuropalliative care physician/educator). This process led to the addition of neurology-specific didactic topics (e.g., brain death) and the separation of hospice referral from end-of-life care. The survey was piloted (step 7) with a small group of neurology residency associate program directors to avoid depleting the analytic sample; minor revisions for clarity were incorporated.

The final instrument (eAppendix 1) comprised 24 items: 1 screening item, 6 questions about program characteristics, 6 about institutional and faculty palliative care resources, 3 about didactic content, 4 about clinical exposure, 3 about the perceived importance of palliative care training, and 1 about barriers to implementation. Two multipart items asked respondents to (1) indicate whether specific didactic topics were covered and (2) rate the importance of 14 domains of palliative care content. These domains were derived from national palliative care scope-of-practice guidelines^1^ and adapted for neurology-specific relevance.^19^

### Sample Assembly

We identified the sampling frame using publicly available directories from the ACGME, Fellowship and Residency Electronic Interactive Database Access, and the United Council for Neurologic Subspecialties. Movement disorder PDs were identified through an unofficial national movement disorder fellowship database maintained by an academic movement disorder PD, given the lack of ACGME-accredited or UCNS-accredited fellowship programs in this subspecialty. Eligible participants included directors of adult and pediatric neurology residencies and subspecialty fellowships with a high expected relevance to palliative care (vascular, epilepsy, multiple sclerosis/neuroimmunology, neurohospitalist, behavioral neurology, geriatric neurology, neurocritical care, neuromuscular, neuro-oncology, and movement disorders). The authors selected neurology subspecialty fellowships for inclusion in the survey based on their collective expertise and assessment of the degree to which neuropalliative care skills are pertinent to clinical practice within each subspecialty.

### Survey Distribution and Administration

The survey instrument was distributed using REDCap, a web-based data management platform.^27^ Using REDCap's automated distribution function, we invited PDs to participate in January of 2025, with 2 additional reminder emails sent later in January and February to nonresponders. Participation was voluntary, and responses were anonymous. The survey introduction outlined the study's purpose and the implied consent for completion. Because there was initially no way to obtain information about movement disorder programs, the survey was distributed to movement disorder PDs in January 2026 when the team gained access to a specialized listserv.

### Data Analysis

Analysis was performed using Stata version 18.^28^ Binary and categorical variables are presented using frequencies and percentages. Likert scales are presented as medians with interquartile ranges.

### Standard Protocol Approvals, Registrations, and Consents

This study was reviewed by the Weill Cornell institutional review board and deemed exempt.

### Data Availability

Anonymized data not published within this article will be made available by request from any qualified investigator.

## Results

The survey was emailed to 798 residency and fellowship PDs. The types and number of programs contacted were adult neurology (191), child neurology (81), neuro-oncology (39), movement disorders (72), neuromuscular (57), neurocritical care (86), behavioral neurology (48), vascular neurology (114), epilepsy (98), neurohospitalist (8), and geriatric neurology (4). We received delivery error messages from 21 programs for which we were unable to find alternative information. This included 4 adult neurology, 1 child neurology, 2 neuro-oncology, 1 neuromuscular, 6 neurocritical care, 5 vascular neurology, 1 epilepsy, and 1 geriatric program. Of the 777 programs contacted, 129 responded; 10 were excluded because they did not identify as PDs of eligible neurology training programs, and 1 was excluded for not answering any content questions, yielding a final analytic sample of 118 (15.2% response rate). Forty-three programs were located in the Northeast, 20 in the Midwest, 31 in the South, 23 in the West, and 1 in Puerto Rico. In total, 111 programs were university based, 7 community based, 3 Veterans Affairs, and 2 indicated “other.” The number and response rates for each type of training program are presented in Table 1. Notably, no responses were recorded from multiple sclerosis or geriatric neurology fellowship programs.

**Table 1:** Characteristics of Training Programs Responding to the Survey, According to Hospital Affiliation, Geographic Location, and Program Size

	Adult neurology	Child neurology	Neuro-oncology	Neuro-muscular	Neuro-critical care	Behavioral neurology	Vascular	Epilepsy	Neuro-hospitalist	Movement disorders
Number of respondents										
N (%)	34 (18)	11 (14)	5 (14)	9 (16)	19 (24)	12 (25)	12 (11)	8 (8)	2 (25)	6 (8)
Program affiliation										
University, n (%)	31 (91)	11 (100)	5 (100)	9 (100)	18 (94.7)	11 (91.7)	11 (91.7)	7 (87.5)	2 (100)	6 (100)
Community, n (%)	4 (11.8)	1 (9.1)	0	0	1 (5.3)	1 (8.3)	0	0	0	0
Veterans Affairs, n (%)	1 (2.9)	0	0	0		2 (16.7)	0	0	0	0
Geographic setting										
Urban, n (%)	28 (82.4)	11 (100)	5 (100)	8 (88.9)	17 (89.5)	9 (75)	10 (83.3)	7 (87.5)	2 (100)	4 (66.7)
Suburban, n (%)	8 (23.5)	1 (9.1)	0	2 (22.2)	4 (21.1)	3 (25)	0	1 (12.5)	0	2 (33.3)
Rural, n (%)	2 (5.9)	0	0	0	1 (5.3)	0	1 (8.3)	1 (12.5)	0	0
Region										
North-east, n (%)	16	3	2	3	6	3	5	3	0	2
Mid-west, n (%)	4	0	1	1	4	3	2	2	1	2
South, n (%)	9	4	0	4	6	3	3	0	0	2
West, n (%)	5	3	2	1	3	3	2	3	1	0
Puerto Rico, n	0	1	0	0	0	0	0	0	0	0
Program size										
1–2, n (%)	0	4 (36)	5 (100)	8 (89)	14 (74)	11 (92)	8 (67)	7 (88)	2 (100)	5 (83.3)
3–4, n (%)	5 (15)	7 (64)	0	0	5 (26)	1 (8)	4 (33)	1 (13)	0	1 (16.7)
5–6, n (%)	8 (24)	0	0	1 (11)	0	0	0	0	0	0
7–8, n (%)	0	0	0	0	0	0	0	0	0	0
9–10, n (%)	0	0	0	0	0	0	0	0	0	0
11 +, n (%)	8 (24)	0	0	0	0	0	0	0	0	0

### Palliative Care Presence in the Program

Of the 118 programs, 117 (99%) indicated that their institutions have a palliative care presence, with 1 respondent unsure. Palliative care was inpatient in 113 (97%) institutions, ambulatory in 79 (68%), home based in 33 (28%), and other in 3 (3%). Twenty respondents (17%) indicated that at least 1 trainee obtained hospice and palliative medicine fellowship training either before or after completing their training program in the past 10 years: 12 from adult neurology, 2 from pediatric neurology, 1 from neuromuscular, 3 from neurocritical care, 1 from vascular, and 1 from epilepsy programs. Twenty-four respondents (20%) indicated that there were faculty in their training program board certified in hospice and palliative care medicine.

### Palliative Care Didactics in the Curriculum

In total, 85/116 (73%) programs reported including palliative care topics in their curricula: 30/33 (91%) adult neurology residency programs and 55/83 (66%) fellowship programs. Topics are grouped into categories: symptom management, interdisciplinary care, goals of care and ethics, and end-of-life care. Tables 2–5 present the percentage of programs that cover each palliative care topic, as well as the perceived importance of each topic to program trainees across the 4 categories. In brief, prognostication (89%), breaking bad news (87%), advanced care planning and goals of care (83%), ethical dilemmas in neurologic care (81%), and psychiatric symptom management (81%) were the most commonly covered topics across all programs. Spiritual care (27%), caregiver support (47%), and opioid pain management (48%) were covered in fewer than 50% of all programs. The perceived importance of topics varied by program type, although breaking bad news, brain death, end-of-life care, and managing ethical conflicts were considered highly important across most program types. Didactic content was taught by physicians dual trained in neurology and hospice and palliative care medicine in 33 (40%) programs, physicians trained in neurology but not in hospice and palliative medicine in 58 (70%) programs, physicians trained in hospice and palliative medicine but not neurology in 39 (47%) programs, physicians trained in neither in 8 (10%) programs, and nonphysicians, such as social workers, chaplains, or nurses, in 22 (27%) programs.

**Table 2:** Frequency of Symptom Management Topics Taught Among Neurology Training Programs and the Perceived Importance of Each Topic, as Rated by Respective Program Directors

Symptom management education											
Component of palliative care											
Training program type	Total (116)	Neurology residency (34)	Pediatric neurology residency (11)	Neuro-oncology (5)	Neuro-muscular (9)	Neuro-critical care (19)	Behavioral neurology (12)	Vascular (11^a^)	Epilepsy (8)	Neuro-hospitalist (1^a^)	Movement disorders (6)
Opioid pain management											
Didactics, (%)	48.2	59.3	81.8	40	44.4	68.4	16.7	36.4	0	100	16.7
Importance (median [IQR])	4 (3–6)	5 (3–5)	5 (3–5)	5 (3–6)	3.5 (2.5–6.5)	7.5 (5–9)	2.5 (1–3)	5 (3–5)	1 (1–2)	5	1.5 (1–2.75)
Nonopioid pain management											
Didactics, (%)	67.5	87.5	81.8	80	88.9	68.4	33.3	45.5	12.5	100	66.7
Importance	7 (5–8)	8 (7–9)	8 (7–10)	7 (3–8)	8 (6.5–8.5)	7.5 (5–9)	3 (2–4)	6 (5–9)	1 (1–4)	5	5 (5–5.5)
Nonpain symptom management											
Didactics, (%)	68.4	78.1	81.8	80	88.9	47.4	83.3	36.4	25	100	100
Importance	7 (5–9)	7 (6–9)	7 (5–9)	9 (6–9)	8.5 (6–10)	5 (5–8)	8 (5.5–9.5)	6 (3–9)	5 (1.5–7)	5	10 (10–10)
Psychiatric symptom management											
Didactics, (%)	80.9	87.9	90.9	100	55.6	57.9	100	54.5	100	100	100
Importance	8 (6–9)	8 (6–9)	7 (5–9)	9 (6–9)	8.5 (6–10)	5 (5–8)	8 (5.5–9.5)	6 (3–9)	5 (1.5–7)	5	9.5 (9–10)

Discordances between didactic provision and perceived importance are indicated with an asterisk, defined by median rank importance of 7 or greater and provision of didactic lecture in less than 50% of reporting programs.

a One vascular PD and 1 neurohospitalist PD did not respond to this field of questions; thus, our total N for these questions was 116.

**Table 3:** Frequency of Interdisciplinary Care Topics Taught Among Neurology Training Programs and the Perceived Importance of Each Topic, as Rated by Respective Program Directors

Interdisciplinary care education											
Component of palliative care											
Training program type	Total (116)	Neurology residency (34)	Pediatric neurology residency (11)	Neuro-oncology (5)	Neuromuscular (9)	Neuro-critical care (19)	Behavioral neurology (12)	Vascular (11^a^)	Epilepsy (8)	Neuro-hospitalist (1^a^)	Movement disorders (6)
Caregiver and family support											
Didactics, (%)	46.5*	34.4	45.5	80	55.6	21.1	100	27.3	37.5	0	100
Importance	7 (5–9)*	6 (5–8)	6.5 (5–8)	8.5 (6–9.5)	5 (3.5–8)	6 (4–8)	9.5 (8–10)	6 (4–10)	5 (4–7)	5	9 (8.5–9)
Spiritual care											
Didactics, (%)	27.4	28.1	10	20*	44.4	26.3	33.3*	18.2	25	0	50
Importance	5 (4–7)	5 (4–7)	5 (5–6)	7 (3–8)*	5.5 (5.5–8.5)	5.5 (4–9)	7.5 (5–8.5)*	5 (3–6)	1.5 (1–4)	5	7.5 (6.5–8.25)
Hospice referral and end of life care											
Didactics, (%)	67.3	75	40	100	77.8	80	66.7	36.4	25	100	100
Importance	7 (5–9)	7 (5–8)	5 (4–8)	10 (7–10)	6.5 (4.5–8.5)	7 (3–9)	8.5 (6–9.5)	6 (5–8)	3 (2.5–3.5)	7	9.5 (9–10)

Discordances between didactic provision and perceived importance are indicated with an asterisk, defined by median rank importance of 7 or greater and provision of didactic lecture in less than 50% of reporting programs.

a One vascular PD and 1 neurohospitalist PD did not respond to this field of questions; thus, our total N for these questions was 116.

**Table 4:** Frequency of Topics Relating to Goals of Care Discussions and Ethical Issues Taught Among Neurology Training Programs and the Perceived Importance of Each Topic, as Rated by Respective Program Directors

Goals of care and ethics education											
Component of palliative care											
Training program type	Total (116)	Neurology residency (34)	Pediatric neurology residency (11)	Neuro-oncology (5)	Neuro-muscular (9)	Neuro-critical care (19)	Behavioral neurology (12)	Vascular (11^a^)	Epilepsy (8)	Neuro-hospitalist (1^a^)	Movement disorders (6)
Advance care planning and GOC discussions											
Didactics, (%)	82.5	90.6	54.5	100	88.9	89.5	91.7	54.5	62.5	100	100
Importance	9 (6–10)	8 (7–10)	8 (6–8)	10 (8–10)	8.5 (7–9.5)	9.5 (8–10)	9.5 (9–10)	7 (5–9)	5 (3–5)	7	10 (9.75–10)
Prognostication											
Didactics	88.6	93.8	72.7	80	77.8	100	91.7	81.8	87.5	100	83.3
Importance	9 (7.25–10)	9 (8–10)	10 (8–10)	9 (9–10)	7.5 (5–9)	10 (9–10)	8 (7.5–9)	9 (8–10)	6 (4–7.5)	10	7.5 (5.75–9.25)
Managing ethical conflicts											
Didactics, (%)	80.7	90.6	81.8	80	44.4	89.5	91.7	45.5*	87.5	100	83.3
Importance	9 (7–10)	9 (8–10)	9.5 (8–10)	7 (4–8)	6.5 (5.5–8.5)	10 (8–10)	8.5 (7.5–9)	8 (6–10)*	7 (5–9)	7	8 (7.25–8.25)
Breaking bad news											
Didactics, (%)	87	100	81.80	100	88.90	89.50	91.70	63.60	50	100	83.3
Importance	9 (8–10)	10 (9–10)	9.5 (9–10)	10 (9–10)	9 (6–10)	9.5 (7–10)	9 (8–10)	9 (7–10)	6 (3–7.5)	7	8.5 (8–9)

Abbreviation: PD = program director.

Discordances between didactic provision and perceived importance are indicated with an asterisk, defined by median rank importance of 7 or greater and provision of didactic lecture in less than 50% of reporting programs.

a One vascular PD and 1 neurohospitalist PD did not respond to this field of questions; thus, our total N for these questions was 116.

**Table 5:** Frequency of Topics Relating to Care of the Imminently Dying Taught Among Neurology Training Programs and the Perceived Importance of Each Topic, as Rated by Respective Program Directors

Care of the imminently dying education											
Training program type											
Component of palliative care	Total (116)	Neurology residency (34)	Pediatric neurology residency (11)	Neuro-oncology (5)	Neuro-muscular (9)	Neuro-critical care (19)	Behavioral neurology (12)	Vascular (11^a^)	Epilepsy (8)	Neuro-hospitalist (1^a^)	Movement disorders (6)
Withdrawal of life-sustaining therapy											
Didactics, (%)	57	75.8	45.5*	40	44.4	94.7	16.7	40*	12.5	100	50
Importance	7 (5–9)	8 (7–9)	7.5 (6–10)*	4 (3–5)	5 (3–7.5)	9.5 (9–10)	3.5 (1.5–7.5)	8 (7–9)*	3 (1–3)	10	1.5 (1–3.25)
Brain death											
Didactics, (%)	59.3	100	100	0	22.2	100	33.3	63.6	37.5	100	0
Importance	9 (3.5–10)	10 (9–10)	10 (9–10)	3 (2–4)	1.5 (1–4.5)	10 (10–10)	2 (1–6.5)	9 (7–10)	3 (1–4.5)	10	1 (1–1.25)
End-of-life care											
Didactics, (%)	59.3	67.7	45.5	100	66.7	68.4	66.7	45.5*	0	100	50
Importance	8 (5–9)	8 (7–10)	6 (5–10)	9 (8–10)	5 (4.5–7.5)	9 (7–9)	7.5 (6–9)	8 (7–9)*	3 (1–4.5)	10	4.5 (1.75–7.75)

Abbreviation: PD = program director.

Discordances between didactic provision and perceived importance are indicated with an asterisk, defined by median rank importance of 7 or greater and provision of didactic lecture in less than 50% of reporting programs.

a One vascular PD and 1 neurohospitalist PD did not respond to this field of questions, thus our total N for these questions was 116.

### Learner Participation in Palliative Care Rotations

Trainees participated in palliative care rotations or clinics in 66 (60%) programs, with 57 (50%) as electives, 8 (7%) as requirements, and 1 (1%) as both. Five (15%) adult residency programs required a palliative care rotation, 2 neuro-oncology programs (40%), 1 neurocritical care program (5%), and 1 behavioral neurology program (8%). The most common palliative care rotation was inpatient general palliative care consults, offered as an elective in 67 (60%) programs and required in 4 (4%), followed by ambulatory general palliative care clinics, offered as an elective in 33 (31%) and required in 2 (2%), and neuropalliative care clinics or consults, offered as an elective in 29 (27%) programs and required in 8 (7%). Inpatient palliative care unit rotations were offered as an elective in 29 (27%) programs, and home-based palliative care or hospice rotations were offered as an elective in 15 (14%) programs. Five respondents reported that trainees participated in other types of palliative care rotations, although they did not provide specifics. Of note, several PDs reported not offering clinical experiences in palliative care but did select offering specific electives, such as consults or neuropalliative care. In programs that require an inpatient palliative care rotation, the total time required was 1–2 weeks for 5/9 respondents, less than 1 week for 2/9, and 3–4 weeks for 2/9. Fifty-seven respondents reported the percentage of trainees who participate in a palliative care elective. In total, 49% reported less than 10%, 25% reported 10%–25%, 14% reported 26%–50%, 9% reported 51%–75%, and 4% reported greater than 75%.

### Importance of Palliative Care Training

When asked how much they agree that palliative care training is important for achieving clinical competency in their subspecialty, on a scale from 1 (strongly disagree) to 10 (strongly agree), the median rank among survey respondents was 7/10, and the IQR was 5–8. By program type, the median and IQR were 7 (6.5–8) for adult neurology, 7 (7–8) for pediatric neurology, 9 (6–9) for neuro-oncology, 6 (4.5–6) for neuromuscular, 7 (6–8) for neurocritical care, 7 (4–8.25) for behavioral neurology, 7 (6–8) for vascular neurology, 5 (3.75–5.25) for epilepsy, 7.5 (7.25–7.75) for neurohospitalist, and 7.5 (6.5–8.5) for movement disorder programs.

### Preparedness of Trainees for Primary Palliative Care Practice

When asked how prepared graduates of their training program are to meet the palliative care needs of their patients, on a scale from 1 (not prepared) to 10 (extremely prepared), the median rank was 6, with an IQR of 5–7. By program type, the median and IQR were 6 (5–7) for adult neurology, 5 (5–6) for pediatric neurology, 7 (7–8) for neuro-oncology, 5.5 (5–6.75) for neuromuscular, 6 (4.75–7.25) for movement disorders, 7 (6–8) for neurocritical care, 7 (4.5–7) for behavioral neurology, 7 (6–7) for vascular neurology, 5 (3.5–5) for epilepsy, and 6.5 (5.75–7.25) for neurohospitalist. When asked how prepared graduates in the subspecialty are nationally to meet patients' palliative care needs, scores were slightly lower, with a median of 5 (IQR 5–7). By program type, the median and IQR were 6 (5–7) for adult neurology, 5 (5-5) for pediatric neurology, 7 (6.75–7.75) for neuro-oncology, 5 (5–6) for neuromuscular, 4 (3–5.5) for movement disorders, 6 (5–8) for neurocritical care, 5 (3.5–6) for behavioral neurology, 6 (6–7) for vascular neurology, 3 (3–4) for epilepsy, and 5 (5-5) for neurohospitalist.

### Barriers to Providing Training in Palliative Care

Forty-five (38%) respondents reported barriers to providing palliative care education within their training program: 56% of adult neurology residency programs and 31% of fellowship programs. Inadequate trainee time, insufficient teaching faculty, and insufficient faculty time were the most commonly reported barriers (Table 6).

**Table 6:** Frequency of Reported Barriers to Teaching Palliative Care Topics in Neurology Residency and Fellowship Programs

Inadequate trainee time, (%)	29 (64)
Lack of adequate teaching faculty, (%)	25 (56)
Inadequate faculty time, (%)	19 (42)
Lack of trainee interest, (%)	15 (33)
Lack of curricula or education resources, (%)	13 (29)
Lack of emphasis on topic by accrediting bodies, (%)	13 (29)
Lack of adequate sites for clinical rotations, (%)	7 (16)
Other, (%)	6 (13)
Other program faculty do not support this training, (%)	5 (11)

## Discussion

Our survey data demonstrate that neurology training programs, both core residency and fellowship programs, continue to exhibit considerable variability in the extent to which they incorporate palliative care education into their curricula.^15^ Nearly all programs reported that there is some palliative care presence in their institutions. Still, variability exists in the number and focus of palliative care topics taught, the skills emphasized, the methods of teaching, and the clinical exposure.

The most notable difference between adult neurology residency programs and fellowship programs was the inclusion of palliative care didactic education. Our study demonstrated 91% of neurology residency programs incorporating this training, which is an improvement compared with the 80% of adult residency programs reported by Mehta et al. in 2018.^15^ This improvement in neuropalliative care educational effort in residency programs may be related to the 2019 ACGME recommendations for neurology residents to receive palliative care education and the development and availability of dedicated neuropalliative care curricula such as EPEC-N, communication skills workshops, and coaching curricula.^19,21,22^ This may reflect uptake of such curricula, although further study is needed to understand which resources are used to implement neuropalliative care education. In comparison, only 66% of fellowship programs report including didactic education in palliative care. This is despite fellowship directors largely agreeing with residency PDs that palliative care training is important for achieving clinical competency in their subspecialty. This gap presents an opportunity to expand palliative care education in subspecialty fellowship programs where palliative care topics are especially pertinent.

In our study, we also note that there seems to be some disagreement as to which topics are considered “palliative” in nature. Several programs “without palliative care curricula” do teach topics, such as breaking bad news and prognostication, suggesting that reporting rates for this education are affected by disparate definitions of “palliative care education.” For example, 2/6 movement disorder programs and 3/9 neuromuscular programs report not having palliative care didactics, despite providing didactics on several of the topics included in the survey. This may also reflect the intertwined nature of palliative care and neurology. Although our field offers opportunities for curative treatment, much of our clinical work focuses on symptom management and quality-of-life optimization.

Even among topics considered to be of moderate or high importance for patient care, there is some discordance in how often they are taught. This is in line with Mehta et al.^15^ (2018), in which 42% of neurology PDs reported dissatisfaction with their own palliative care education curricula. This is especially true for skills commonly shared among an interdisciplinary team. For example, caregiver support had a median importance ranking of 7 of 10 among all survey respondents. However, only 46.5% of programs provided formal education on this topic. Similarly, spiritual support had an overall median importance ranking of 5, although only 27.4% of programs provided didactics on the subject. One method of addressing this discordance may be to collaborate with interdisciplinary professionals such as social work and chaplaincy for didactic content, noted in only 27% of programs in our survey, which would also increase exposure to a team-based care approach.

Our survey results highlight the specialty-specific nature of these neuropalliative care skills. These differences, as reported by PDs, may help to identify priorities for growth in subspecialty-specific neuropalliative care education. For example, the discordance in perceived importance and didactic content on caregiver support was most notable in adult neurology residency programs and neurocritical care and vascular neurology fellowship programs. Vascular neurology PDs ranked several palliative care topics as highly important, including determination of brain death, end-of-life care, and withdrawal of life-sustaining treatments, but a minority of programs report providing dedicated didactic material on these topics. The ranking of importance and the provision of didactic content on specific neuropalliative care topics seem to align with known differences in the clinical responsibilities of neurologic subspecialties. For example, neurocritical care PDs ranked “withdrawal of life-sustaining treatment” a median of 9.5 in importance. By contrast, epilepsy fellowship directors rated this skill set a 3, and neurology residency PDs ranked this topic a median importance of 8 of 10. These results correspond to the varying clinical demands of these specialties. This broad survey may therefore allow subspecialty training programs to assess their own neuropalliative care curricula relative to similar programs and to identify priorities for improving training in these areas.

The most frequently noted barrier to providing palliative care training was inadequate trainee time. As our understanding of the pathophysiology, diagnosis, and management of neurologic disease advances, neurology trainees have more content to master.^13,14^ Training programs therefore face an increasing challenge in prioritizing trainee time, didactic education, and clinical exposure. One option for reducing this barrier may be to incorporate palliative care training into existing local didactic and simulation curricula. For example, a simulation session focused on delivering an amyotrophic lateral sclerosis diagnosis may provide trainees with the opportunity to learn and solidify their understanding of the disease and its prognosis, while also offering supervised practice and feedback on skills in serious illness communication. By integrating both medical knowledge and serious illness communication skills practice, programs may more easily overcome time-related barriers.

Another common barrier to palliative care education is the availability of appropriate teaching faculty within the neurology department. One solution could be to leverage non-neurology faculty trained in palliative care, as well as other staff (social workers, chaplains, nurses), through required palliative care rotations and a wider availability of electives. Although general palliative care rotations may not always be neurology-focused, the skills modeled and acquired through these rotations are certainly relevant to neurologic practice. Palliative care programs are also noted to face shortages of teaching faculty and resources, so partnering with palliative care faculty may be fruitful to optimize the time spent and skills learned by trainees. To further mitigate staffing issues and problems with faculty expertise and availability, programs may also consider using existing freely available neuropalliative care curricula such as EPEC-N, developed to enhance the delivery of neuropalliative care education nationally, and which does not require local subspecialty expertise.^22^

There are limitations to our study. The relatively low overall response rate (15.2%), combined with the absence of responses from multiple sclerosis (MS)/neuroimmunology and geriatric neurology programs, limits the generalizability of our findings, particularly for subspecialties with few or no respondents. Programs that responded may disproportionately represent those with greater existing interest in neuropalliative care education, potentially inflating estimates of curriculum prevalence and perceived importance. In addition, movement disorder fellowship directors were surveyed approximately 1 year after other PDs because of the absence of a publicly accessible directory for this subspecialty. At the same time, this reflects a pragmatic sampling constraint. Temporal differences in data collection may limit direct comparisons between movement disorder programs and other subspecialties. Finally, variability in the construct of palliative care itself, and its boundaries with general neurology practice vis-a-vis topics such as breaking bad news, may have artificially deflated the reported prevalence of palliative care training.

This study has several strengths, including its broad sampling across residency and multiple fellowship training levels and subspecialties, which provides a national overview of neuropalliative care education and highlights subspecialty-specific variability. Further work is needed to determine optimal pedagogical approaches for training in neuropalliative care, particularly because these needs vary across subspecialties. Because the burden of serious neurologic illness continues to grow, training programs have an opportunity, and an obligation, to close the gap between the palliative care needs of their patients and the preparation of their graduates.

## Glossary

AAN: Academy of Neurology
ACGME: Accreditation Council for Graduate Medical Education
IQR: interquartile range
PD: program director
